# Genetic removal of synaptic Zn^2+^ impairs cognition, alters neurotrophic signaling and induces neuronal hyperactivity

**DOI:** 10.3389/fneur.2022.882635

**Published:** 2023-01-20

**Authors:** Emily C. Vogler, Matthew Mahavongtrakul, Kristianna Sarkan, Ryan C. Bohannan, Silvina Catuara-Solarz, Jorge Busciglio

**Affiliations:** ^1^Department of Neurobiology and Behavior, University of California, Irvine, Irvine, CA, United States; ^2^Institute for Memory Impairments and Neurological Disorders, University of California, Irvine, Irvine, CA, United States; ^3^Center for the Neurobiology of Learning and Memory, University of California, Irvine, Irvine, CA, United States

**Keywords:** Alzheimer's disease (AD), synaptic zinc, neurodegeneration, neuronal hyperactivity, hippocampus, neurotrophic signaling, ZnT3, zinc transporter

## Abstract

Vesicular Zn^2+^ (zinc) is released at synapses and has been demonstrated to modulate neuronal responses. However, mechanisms through which dysregulation of zinc homeostasis may potentiate neuronal dysfunction and neurodegeneration are not well-understood. We previously reported that accumulation of soluble amyloid beta oligomers (AβO) at synapses correlates with synaptic loss and that AβO localization at synapses is regulated by synaptic activity and enhanced by the release of vesicular Zn^2+^ in the hippocampus, a brain region that deteriorates early in Alzheimer's disease (AD). Significantly, drugs regulating zinc homeostasis inhibit AβO accumulation and improve cognition in mouse models of AD. We used both sexes of a transgenic mouse model lacking synaptic Zn^2+^ (ZnT3KO) that develops AD-like cognitive impairment and neurodegeneration to study the effects of disruption of Zn^2+^ modulation of neurotransmission in cognition, protein expression and activation, and neuronal excitability. Here we report that the genetic removal of synaptic Zn^2+^ results in progressive impairment of hippocampal-dependent memory, reduces activity-dependent increase in Erk phosphorylation and BDNF mRNA, alters regulation of Erk activation by NMDAR subunits, increases neuronal spiking, and induces biochemical and morphological alterations consistent with increasing epileptiform activity and neurodegeneration as ZnT3KO mice age. Our study shows that disruption of synaptic Zn^2+^ triggers neurodegenerative processes and is a potential pathway through which AβO trigger altered expression of neurotrophic proteins, along with reduced hippocampal synaptic density and degenerating neurons, neuronal spiking activity, and cognitive impairment and supports efforts to develop therapeutics to preserve synaptic zinc homeostasis in the brain as potential treatments for AD.

## 1. Introduction

Zinc is one of the most abundant trace elements in the brain, with its distribution and levels in the central nervous system tightly regulated by various proteins, including ZnT3, which transports Zn^2+^ into presynaptic vesicles of glutamatergic neurons in the mossy fiber tract of the hippocampus (1, 2). This synaptic pool of Zn^2+^ is released with glutamate and modulates neurotransmission, influencing different ion channels and receptors (3–6). Dysregulation of zinc homeostasis has been implicated in the pathology of Alzheimer's disease (AD). Drugs targeting zinc in the brain have been shown to inhibit amyloid beta (Aβ) accumulation (7), reduce Aβ oligomer (AβO) levels, and restore cognition (8, 9), dendritic spine density, and synaptic protein levels (10) in mouse models of AD. AβO also bind and sequester Zn^2+^, resulting in synaptic loss (11) and inhibition of long-term potentiation (12) in rat hippocampus. Zn/AβO inhibit hippocampal long-term potentiation (LTP) and activate hippocampal microglia, while removal of Zn^2+^ leads to restoration of Zn/AβO to Aβ fibrils (13). In humans, non-demented subjects with AD neuropathology have lower total zinc levels and preserved ZnT3 expression compared to AD patients, implicating dysregulation of synaptic Zn^2+^ in cognitive impairment (14). Zn^2+^ and ZnT3 have been implicated in other neurodegenerative disorders, including Parkinson's disease (15) and Huntington's disease (16). Zinc depletion has been found to promote expression of the apoptotic protein Caspase-3 (17), reduce adult hippocampal neurogenesis (18), and negatively affect memory, expression of proteins critical to neuronal health and synaptic function (19).

We have previously reported that the accumulation of AβO in synapses is regulated by synaptic activity and enhanced by the presence of synaptic Zn^2+^. Furthermore, we found that AβO colocalize with N-methyl-D-aspartate receptor (NMDAR) NR2B subunits and that synaptic accumulation of AβO correlates with synaptic loss in AD brains (20). AβO has also been demonstrated to induce apoptotic cell death through interaction with NR2B-containing NMDARs in hippocampal cultures (21). Zn^2+^ is a potent inhibitor of NMDAR responses (22–27), which is dependent on NMDAR subunit composition (28–34). Zn^2+^ has been shown to diffuse beyond the synaptic cleft and inhibit extrasynaptic NMDARs (6) and inhibit postsynaptic NR2A NMDARs (35), strongly suggesting that the interaction between Zn^2+^ and AβO disrupts NMDAR signaling.

Genetic elimination of *ZnT3* results in the absence of Zn^2+^ in synaptic vesicles and enhanced susceptibility to induced seizures (36, 37). Similarly, Zn^2+^ chelation induces hyperexcitability in hippocampus (38), and infusing Zn^2+^ into the hippocampus delays the development of seizures in kindling models of epilepsy (39). In addition to inhibiting NMDARs, Zn^2+^ activates TrkB receptors (3, 40) and regulates activation of Erk1/2 (17, 41). Consequently, AβO sequestration of Zn^2+^ at synaptic sites may trigger neuronal hyperactivity and neurodegenerative pathways (42). Disruption of neuromodulation by synaptic Zn^2+^ is modeled in a transgenic *ZnT3* knockout (ZnT3KO) mouse model. Though cognitively normal at a young age, ZnT3KO mice exhibit increased electroencephalogram (EEG) spiking activity after kainate-induced seizures (37) and develop age-dependent cognitive impairment and loss of synaptic and neurotrophic proteins similar to those found in AD (43).

Due to an increase in aging populations and little progress with therapeutics to effectively target this impaired cognition, there is an immediate need for understanding the relationship between neurodegeneration and biometals. To further understand the effects of disrupting Zn^2+^ neuromodulation, we profiled the progression of impairment in hippocampal-dependent memory as ZnT3KO mice age and we assessed NMDA signaling, Erk phosphorylation, neuronal spiking activity, and markers of epileptiform activity and neurodegeneration. We found a progressive decline in cognition, reduced activity-dependent increases in Erk phosphorylation and brain-derived neurotrophic factor (BDNF), and differential regulation of NMDAR subunits, along with increased neuronal spiking and alterations consistent with increasing epileptiform activity and neurodegeneration. These findings demonstrate that synaptic Zn^2+^ is critical in normal neuronal function and that disruption of Zn^2+^ neurotransmission triggers progressive, age-dependent alterations consistent with those triggered by AβO-mediated AD pathology.

## 2. Materials and methods

### 2.1. Experimental design and statistical analysis

ZnT3KO mice are reported to initially have normal cognition that becomes impaired with age, indicating that the lack of synaptic zinc is not the direct cause of impaired cognition, but induces neurodegenerative effects leading to impairment. Consequently, we sought to investigate the effects of disruption of Zn^2+^ modulation of neurotransmission in cognition, protein expression and activation, and neuronal excitability in ZnT3KO mice. Basal biochemical and activity-dependent alterations in neurotrophic NMDAR signaling and BDNF mRNA expression in ZnT3KO mice before the onset of cognitive deficits were assessed in acute hippocampal slice cultures from juvenile WT and ZnT3KO mice. Neuronal hyperactivity before the onset of cognitive deficits in ZnT3KO was assessed using EEG recordings in young WT and ZnT3KO. Progressive age-dependent cognitive decline, neurodegenerative protein expression, and neurodegenerative alterations in both biochemistry and brain cytoarchitecture associated with epileptiform activity were assessed in cohorts of aged WT and ZnT3KO at 3, 6, 12, and 15 months of age. All animal experiments were approved by the University of California, Irvine Institutional Animal Care and Use Committee and conformed to all University and USDA animal care regulations. Homozygous male and female 129Sv wild-type (WT) and ZnT3KO mice (from Richard Palmiter, University of Washington) were housed in the vivarium at the University of California, Irvine, maintained on a 12-h day/night cycle in separate WT and ZnT3KO colonies, and provided food and water *ad libitum*. After the eighth generation all of the adult mice in the colonies were genotyped, using the Transnetyx genotyping service, to check for genetic drift. For experiments using adult mice, dams and pups from litters born within 5 days of each other were combined in one cage to a maximum of 3 dams and 14 pups until weaning at 21 days of age. The weaned mice were sexed and housed 5 per cage by sex, with equal numbers of males and females. Equal numbers of males and females were used for experiments unless health precluded participation of an individual. For experiments using hippocampal slices from 3 to 4-week-old mice, hippocampal tissue from litters consisting of at least 4 pups were combined as one sample. GraphPad Prism 7 software was used for statistical analysis. Data is presented as mean ± SEM, with raw data from two compared conditions analyzed by two-tailed Student's *t*-test, reporting *t*-test statistics (t), degrees of freedom (df), and *p*-value (*p*) in the results.

### 2.2. Object location memory (OLM)

OLM, also referred to as “novel place learning” or “novel object location,” is a memory task that assesses rodents' preference for novelty; in this case, the novelty of the object is placement in a different location (44). Mice were handled for 2 min for 5 consecutive days and habituated to the testing arena for 5 min for 6 consecutive days, with days 4 and 5 of handling overlapping with days 1 and 2 of habituation. On training day, two identical objects were introduced into the training area, spaced equally apart. Animals were allowed to explore the objects for 10 min to become familiarized with the objects and their location. After 24 h, the mice were tested for long-term object location memory by returning the mice to the testing arena with one of the previous objects placed in the same location and the other object placed in a novel location, and allowing the mice to explore the objects. All extra-maze cues in the room were kept constant throughout training and testing. Habituation, training, and testing were all filmed and analyzed in EthoVision software. Exploration of the objects was scored by two investigators blind to subjects' condition. Time of exploration (t) was determined by the subject being within 1 cm of the object and the elongated line from eyes to nose would intersect the object. The discrimination index (DI) was determined by:


$$\begin{array}{l} DI=(t_{novel}t_{familiar})/(t_{novel}+t_{familiar})\times 100\% \end{array}$$

### 2.3. Preparation of hippocampal slices

Three- to four-week-old WT or ZnT3KO mice were used to prepare acute hippocampal slices. Animals were anesthetized with 5% isoflurane and decapitated and their brains were rapidly removed and rinsed with ice-cold artificial cerebrospinal fluid (ACSF). The hippocampus was dissected out and sliced into 350 μm-thick sections with a chilled Stoelting tissue chopper and transferred to individual wells in a 48-well plate containing cold ACSF. The ACSF was replaced with 500 μL of 37°C Neurobasal medium and stabilized at 37°C and 5% CO_2_ for 1 hour before experimental procedures. Slices from two subjects were pooled for each experiment to result in an equal number of slices for each condition in the experiment. Conditions were no treatment, KCl, and KCl with NMDAR subunit-specific antagonists.

### 2.4. Chemical modulation of neuronal activity

Hippocampal slices were incubated with KCl (20 mM, Sigma). The following drugs were used to modulate NMDA receptor activity: NR2A antagonist [(R)-[(S)-1-(4-bromo-phenyl)-ethylamino]-(2,3-dioxo-1,2,3,4-tetrahydroquinoxalin-5-yl)-methyl]-phosphonic acid (PEAQX or NVP-AAM077), Sigma; NR2B antagonist 4-[2-[4-(cyclohexylmethyl)-1-piperidinyl]-1-hydroxypropyl]phenol (ifenprodil), Sigma. Drugs were reconstituted with sterile H_2_O and diluted in Neurobasal media.

### 2.5. Quantification of protein levels after chemical modulation

Hippocampal slices were transferred to RIPA buffer containing 10 μL/mL Halt protease and phosphatase inhibitor cocktail (Thermo Fisher Scientific), homogenized for 20 s with an IKA Ultra-Turrax T8 homogenizer, and centrifuged at 14,000 x g for 1 h. The supernatant was removed and stored at −80°C until immunoblotting. Protein concentration was assayed using the Thermo Fisher Scientific Pierce BCA protein assay kit.

### 2.6. Western blot

Proteins were prepared for SDS-PAGE using the BioRad Criterion system. Twenty micrograms of proteins were equalized with RIPA buffer, diluted 1:1 with Laemmli sample buffer (BioRad)/β-Mercaptoethanol prepared at a ratio of 95:5, heated to 100°C for 5 min, loaded into wells of Criterion 4-20% Tris/HCl 1.0 mm precast gels, and separated by gel electrophoresis for 2 h at 125 mV in BioRad tris/glycine/SDS running buffer. Proteins were then transferred onto PVDF membranes (Immobilon) by Western blot for 3 h at 4°C in Towbin buffer (BioRad) with Precision Plus Protein All Blue Standards (BioRad) to identify molecular weights. Membranes were blocked overnight at 4°C in 5% non-fat milk or 5% BSA with 0.1% Tween 20 in TBS and probed with primary antibodies overnight at 4°C in 2.5% non-fat milk or BSA with 0.05% Tween 20 in TBS. Membranes were then washed three times for 5 min each with 0.1% Tween 20 in TBS and incubated in goat anti-mouse and goat anti-rabbit IgG (H+L) HRP-conjugated secondary antibodies (Thermo Fisher Scientific) in 2.5% non-fat milk or BSA with 0.05% Tween 20 in TBS for 2 h at room temperature. Membranes were washed three times for 5 min each with 0.1% Tween 20 in TBS and incubated for 5 min in SuperSignal West Femto, Dura, or Pico chemiluminescent substrate and imaged on a BioRad ChemiDoc XRS+ imaging system and analyzed for quantification with ImageLab software (BioRad). All data were normalized to GAPDH (glyceraldehyde 3-phosphate dehydrogenase). Primary antibodies used were mouse anti-GAPDH (Abcam 125247), rabbit anti-GAPDH (Abcam 37168), rabbit anti-BDNF (Abcam 6201), rabbit anti-MAPK (ERK1/2; Cell Signaling Technology 4695), rabbit anti-phospho-p44/42 MAPK (ERK1/2; Cell Signaling Technology 9106), rabbit anti-Akt (pan; Cell Signaling Technology 4691), rabbit anti-phospho-Akt (Cell Signaling Technology 2965), and mouse anti-calbindin (Swant 300). Secondary antibodies were Alexa Fluor 488 goat anti-rabbit, Alexa Fluor 488 goat anti-mouse, Alexa Fluor 594 goat anti-rabbit, and Alexa Fluor 594 goat anti-mouse (Molecular Probes).

### 2.7. Dot blot

Twenty micrograms of proteins were equalized with RIPA buffer and loaded into adjacent wells of an S&S manifold-I dot-blot apparatus housing a nitrocellulose membrane. The samples were drawn through the apparatus and into the membrane by vacuum and blocked overnight at 4°C in 5% non-fat milk with 0.1% Tween 20 in TBS and probed with primary antibodies overnight at 4°C in 2.5% non-fat milk with 0.1% Tween 20 in TBS. Antibodies used were rabbit anti-NPY (Abcam 10980) and mouse anti-MAP2 (Millipore MAB4318). Membranes were washed three times for 5 min each with 0.1% Tween 20 in TBS and incubated in goat anti-mouse and goat anti-rabbit IgG (H+L) HRP-conjugated secondary antibodies (Thermo Fisher Scientific) for 2 h at room temperature. Membranes were washed three times for 5 min each with 0.1% Tween 20 in TBS and incubated for 5 min in SuperSignal West Dura chemiluminescent substrate and imaged on a BioRad ChemiDoc XRS+ imaging system and analyzed for quantification with QuantityOne 1-D analysis software (BioRad). All data were normalized to MAP2 adjacent samples.

### 2.8. Extraction of RNA from tissue and purification

Hippocampal slices from 3- to 4-week-old WT or ZnT3KO mice were prepared as previously described for chemical modulation of neuronal activity and incubated in 20 mM KCl or vehicle for 3.5 h at 37°C and 5% CO_2_, transferred to 1 mL of Trizol (Invitrogen 15596-026) and homogenized for 20 s with an IKA Ultra-Turrax T8 homogenizer. RNA was isolated using the Trizol method (Invitrogen) and stored at −80°C until purification using a Qiagen RNeasy mini-kit (Qiagen 74104) with on-column DNase I digestion according to the manufacturer's protocol. RNA concentrations and purity were measured using a NanoDrop 2000 spectrophotometer and analyzed using NanoDrop software (Thermo Fisher Scientific).

### 2.9. RT-PCR and qPCR

cDNA was prepared from up to 1.0 μg RNA using the BioRad iScript Reverse Transcription (RT) Supermix system (BioRad 170-8840) and RT-PCR was performed with a PCR Sprint thermal cycler using the BioRad iScript RT protocol. The resulting cDNA was diluted 1:5 in DNAse/RNAse-free H_2_O and used for qPCR by the SYBR green method (BioRad 170-8880) with a BioRad DNAengine Peltier thermal cycler using the iQ SYBR green protocol. Primer sequences (Eurofins MWG Operon) used were:

TCGTTCCTTTCGAGTTAGCC (mBDNFexS)

TTGGTAAACGGCACAAAAC (mBDNFexAS)

AGGTATCCTGACCCTGAAG (mActinS)

GCTCATTGTAGAAGGTGTGG (mActinAS).

The resulting data was analyzed using MJ Opticon Monitor (BioRad) and mRNA expression was measured with satisfactory reproducibility between triplicates and fold change relative to average ΔCt was calculated as 2^−ΔΔCt^ and normalized to actin.

### 2.10. Immunohistochemistry and image analysis

Free-floating 40 μm-thick sections of 4% paraformaldehyde (PFA)-fixed mouse brain sections were permeabilized and blocked with 5% BSA/0.3% Triton X-100/PBS for one-to-two hours and incubated overnight at 4°C with primary antibodies in 2.5% BSA/0.015% Triton X-100/PBS. Primary antibodies used were mouse anti-calbindin 1:1,000 (Swant 300), rabbit anti-NPY 1:500 (Abcam 10980), mouse anti-GAP-43 1:1,000 (Calbiochem CP09L), rabbit anti-synaptoporin 1:750 (Synaptic Systems 102002), mouse anti-synaptophysin 1:1,000 (Cell Signaling 7H12) and mouse anti-synaptophysin 1:1000 (Abcam ab8049). The sections were washed three times for 5 min each with PBS and incubated for 1 hour at room temperature with secondary antibodies in 2.5% BSA/0.015% Triton X-100/PBS. Secondary antibodies used were goat anti-rabbit and goat anti-mouse fluorescent conjugated Alexa 488 and Alexa 594 1:5,000 (Invitrogen). The sections were washed again three times for 5 min each with PBS and incubated in Hoescht 33342 solution 1:10,000 (Invitrogen) in PBS for 5 min, washed again as before and mounted on a microscope slide with Vectashield mounting media (Vector Laboratories) with a microscope cover glass. Slides were dried for 1 h at 37°C and stored at 4°C.

Images were captured with an Axiovert 200 inverted microscope and processed using AxioVision software (Zeiss) for visualization of calbindin and NPY immunolabeling. An Apotome device (Zeiss) was used for optical “Z” sectioning of multifluorescence signals, then a maximum intensity projection of all z planes was created for analysis of synaptoporin and GAP-43 immunolabeling. This allowed visualization of individual processes through multiple planes of focus.

Calbindin and NPY immunolabeling in specific hippocampal regions were quantified as percent area in two fields from two samples for each condition. The mean fluorescence was measured in each sample in cell-free areas to determine background autofluorescence and the images adjusted to normalize the level of background across green and red channels. Synaptoporin puncta were counted at 63X magnification with three fields imaged for each subject in the molecular layer of the dentate gyrus of ZnT3KO mice and values were expressed as ratio relative to WT puncta for each subject. Puncta were counted if the size and intensity were above a threshold that was determined to be background fluorescence. GAP-43 levels were analyzed in ten fields from each subject imaged at 63X magnification and the percent area of GAP-43 immunofluorescent labeling in each field was quantified using the AxioVision (Zeiss) automated measurement program and the average percent area was calculated.

### 2.11. Fluorojade labeling and image analysis

40 μm-thick PFA-fixed mouse brain sections were mounted on microscope slides with 0.5% gelatin (Sigma) mounting solution and dried for 5 min at 45°C. The slides were then immersed in 100% ethanol for 5 min, 70% ethanol for 2 min, and rinsed in deionized water for 2 min. Slides were then immersed in a solution of 0.06% KMnO_4_ (Sigma) for 10 min with gentle shaking and rinsed in deionized water for 2 min. Slides were then immersed in a solution of 0.0004% Fluorojade B (Millipore) in 0.1% acetic acid for 20 min with gentle shaking in the dark, rinsed in deionized water three times for 1 min each and dried at 45°C for 10 min. Slides were then immersed in xylene for three changes of 1 min each and coverslipped with DPX mountant (Sigma).

Two-to-four images of the CA3 region of the hippocampus were captured at 20X with an Axiovert 200 inverted microscope and processed using AxioVision software (Zeiss).

Background intensity was normalized between samples and Fluorojade puncta were marked if the size and intensity were above a threshold that was determined to be background fluorescence and counted as positive labeling if the Fluorojade puncta was co-localized with Hoescht staining to eliminate non-specific Fluorojade label. The number of Fluorojade- and Hoescht-positive cells counted in the multiple images was averaged to quantify degenerating neurons for each animal.

### 2.12. EEG detection of epileptiform activity

EEG activity was recorded in 2-month-old freely moving mice using a Neurologger recording device (TSE Systems) and processed with Spike2 v8.07 (Cambridge Electronic Design) analysis software. Surgeries were performed as previously described in Vogler et al. (45). Briefly, mice were anesthetized with 3% isoflurane, the scalp removed, then 0.255 mm diameter electrodes were implanted 1 mm deep into 0.26 mm holes drilled into the skull and fastened with cyanoacrylate glue. A mounting pedestal was fashioned with dental cement over the electrode wires and exposed skull, and the wound sealed with cyanoacrylate glue. Two recording electrodes were placed at −1.34 mm A/P, ± 1.5 mm M/L and two at −5.34 mm A/P, ± 3.00 mm M/L relative to bregma and the reference electrode was placed at −7.00 mm A/P, 0.50 mm L relative to bregma. Mice were intraperitoneally injected with 3 mg/kg ketoprofen prior to surgery, provided with 1.25 mg/ml acetaminophen in their drinking water for 3 days post-surgery, and housed individually in a home cage for the duration of the experiment. Mice were briefly anesthetized with isoflurane at least 4 days post-surgery and the Neurologger was affixed to the pedestal. The mice were then returned to their home cage, which was then placed in a grounded Faraday cage for a recording period of 20–26 h. Recordings with poor signal from any electrode were rejected. EEG data during movement detected by the accelerometer function of the Neurologger device was removed manually and the raw EEG data was bandpass filtered from 1 to 80 Hz. Spike threshold was a peak amplitude 2.5X greater than the average baseline to determine spikes/hour.

## 3. Results

### 3.1. While juvenile ZnT3KO mice have normal hippocampal-dependent cognition, there is a progressive cognitive decline as they age

**Figure 1:**
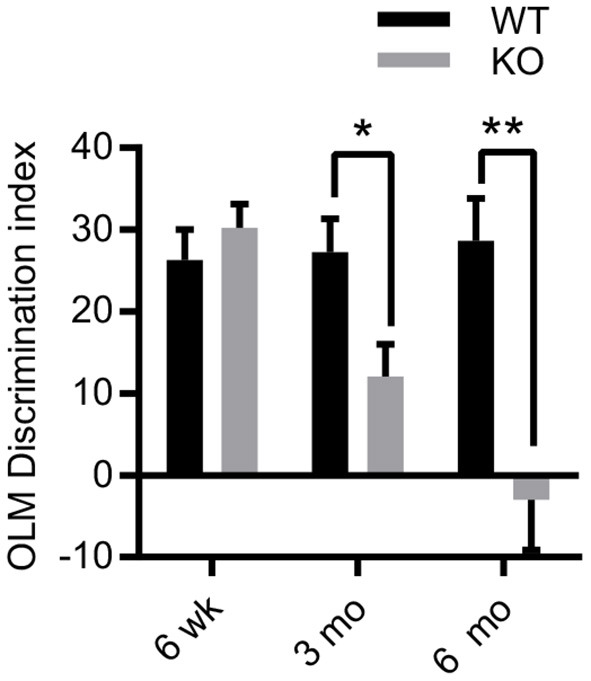
Age-dependent deficits in hippocampal-dependent memory in ZnT3KO mice. Object location memory (OLM) task discrimination index shows age-dependent memory impairment in ZnT3KO mice, becoming significant at 3 months of age and profound by 6 months. WT, wild type, *n* = 8–13; KO, ZnT3KO, *n* = 14–16; DI, discrimination index [(novel-familiar)/(novel+familiar)*100]; *p < 0.05, **p < 0.01. Data were analyzed by two-tailed unpaired *t*-test. Error bars indicate the mean ± SEM.

The Morris water maze task (MWM) has been used to assess memory in ZnT3KO mice by several research groups. Those previous findings indicate that 6-10-week-old ZnT3KO mice are unimpaired (46), while 3-month-old ZnT3KO gave variable results in different studies (43, 47, 48), and 6-month-old ZnT3KO mice exhibit significant impairment (43). As ZnT3 and synaptic Zn^2+^ are heavily concentrated in the hippocampus, we selected a task requiring hippocampal-dependent spatial memory; specifically, the object location memory (OLM) task (44, 49), which has no requirement to learn a task or to introduce a motivating factor. Instead, OLM relies on rodents' inherent preference for novelty over familiar circumstances, including familiar objects placed in novel locations (50, 51), and requires hippocampal function for encoding, consolidation, and retrieval of memory for the location of the objects (52, 53). We profiled hippocampal-dependent memory in ZnT3KO mice at 6 weeks, 3 months, and 6 months of age in both male and females, finding normal cognition at 6 weeks (DI, WT, 26.31 ± 3.714, *n* = 13; KO, 30.26 ± 2.859, *n* = 16), impairment at 3 months (DI, WT, 27.22 ± 4.083, *n* = 9; KO, 12.07 ± 3.93, *n* = 14; t = 2.567 df = 21, *p* = 0.018) and profound impairment at 6 months (DI, WT, 28.63 ± 5.193, *n* = 8; KO, −2.972 ± 6.136, *n* = 14; *t* = 3.487 df = 20, *p* = 0.0023; Figure 1), with no difference between male and females in performance at each age (data not shown). Thus, our results suggest that the lack of synaptic Zn^2+^ does not directly cause cognitive impairment as young ZnT3KO have intact cognition, but does trigger progressive neurodegenerative processes as ZnT3KO age, leading to impaired cognition.

### 3.2. Reduced hippocampal expression and phosphorylation of Erk1/2 in ZnT3KO mice

**Figure 2:**
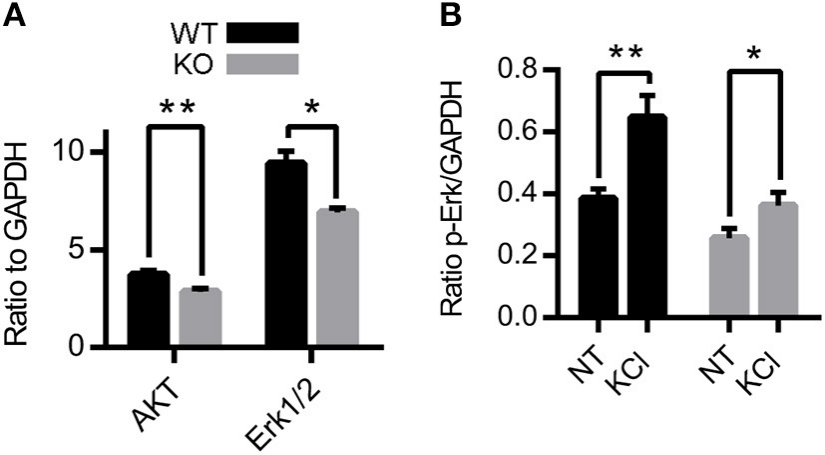
Protein level and activity-dependent phosphorylation of AKT and Erk1/2 are reduced in ZnT3KO hippocampus. **(A)** Basal levels of AKT and Erk1/2 proteins are reduced in ZnT3KO. **(B)** Incubation with 20 mM KCl results in a greater increase in p-Erk1/2 in WT than in ZnT3KO hippocampus. WT, wild type; KO, ZnT3KO; AKT, Erk1/2, *n* = 6; p-Erk1/2, WT, *n* = 4, KO, *n* = 5; NT, no treatment (vehicle); KCl, 10-min incubation in 20 mM KCl; *p < 0.05, **p < 0.01; values expressed as normalized densitometry relative to GAPDH. Data were analyzed by two-tailed unpaired *t*-test. Error bars indicate the mean ± SEM.

Because Zn^2+^ activates TrkB receptors and inhibits NMDA receptors (3, 25, 40, 54), we investigated the effects of the removal of synaptic Zn^2+^ in the expression and activation of two proteins found in both signaling pathways: Erk1/2 and AKT (31, 55–60). Acute hippocampal slices from 3-4-week-old ZnT3KO and WT mice were treated for 10 min with vehicle or 20mM KCl to stimulate vesicle release to assess basal expression and activity-induced phosphorylation levels, followed by protein extraction and analysis by Western blot. Densitometry normalized to GAPDH showed reduced levels of AKT and Erk1/2 in ZnT3KO under basal conditions (Figure 2A and Supplementary Figure 1A). The percent increase of p-AKT after treatment with KCl was similar in both genotypes (data not shown). However, the increase in p-Erk1/2 after treatment with KCl was significant in WT mice (Figure 2B and Supplementary Figure 1B), with a reduced percent increase of p-Erk in ZnT3KO mice (p-Erk1/2 % increase, WT, 72.8 ± 3.9%, *n* = 4; KO, *n* = 5, 41.1 ± 3.2%; *t* = 6.341 df = 7, *p* = 0.0004). Levels of p-Erk1/2 at basal conditions were also reduced in ZnT3KO mice (p-Erk NT, WT, 0.41 ± 0.02, *n* = 4; KO, 0.26 ± 0.02, *n* = 5; *t* = 4.054 df = 7, *p* = 0.0048). These results invited further investigation of the role of synaptic Zn^2+^ in activation of Erk1/2.

### 3.3. Zn^2+^ inhibits NR2A-mediated activation of Erk1/2

**Figure 3:**
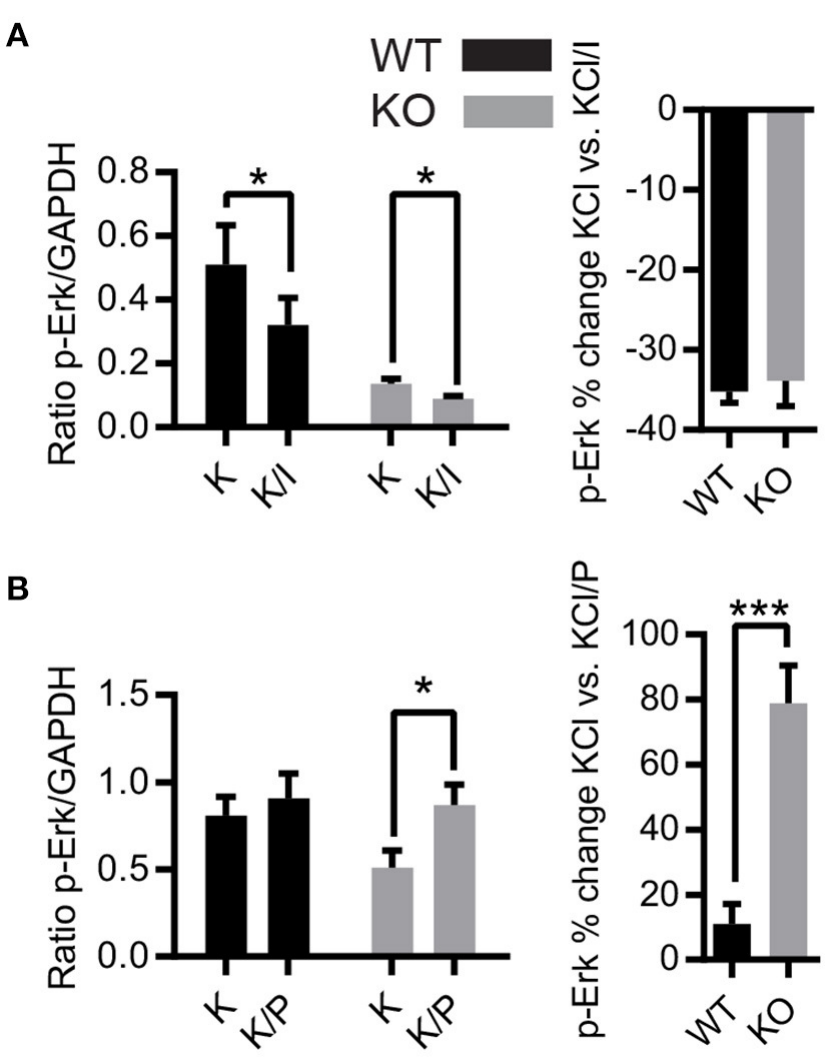
Activity-dependent phosphorylation of Erk1/2 is differentially regulated by NMDAR subunits in ZnT3KO hippocampus. **(A)** Treatment with ifenprodil, an antagonist of the NMDAR NR2B subunit, showed a decrease in activity-dependent Erk1/2 phosphorylation with a similar percent decrease in both WT and ZnT3KO mice. **(B)** The activity-dependent phosphorylation of Erk1/2 is increased by incubation with the NR2A antagonist PEAQX, with a significant percent increase in ZnT3KO. WT, wild type; KO, ZnT3KO; ifenprodil, WT, *n* = 4 KO, *n* = 6; PEAQX, WT and KO, *n* = 5; K, 10-min incubation in 20 mM KCl; K/I, 10-min co-incubation with 20 mM KCl and 50 μM ifenprodil: K/P, 30-min incubation with 0.5 μM PEAQX followed by 10-min co-incubation with 0.5 μM PEAQX/20 mM KCl; *p < 0.05, ***p < 0.0005; values expressed as normalized densitometry relative to GAPDH. Data were analyzed by two-tailed unpaired *t*-test. Error bars indicate the mean ± SEM.

NMDARs have differential effects on the activation of Erk1/2 depending on NMDAR location and subunit composition, but conflicting results have been reported (31, 33, 57). Consequently, we investigated the effects of removal of synaptic Zn^2+^ on differential activation of Erk1/2 by NMDAR subunits using antagonists of NR2A (PEAQX) and NR2B (ifenprodil) NMDAR subunits. Acute hippocampal slices were treated with vehicle, 20 mM KCl, or 20 mM KCl+antagonist and the protein samples were prepared for Western blot. Treatment with KCl+ifenprodil reduced levels of p-Erk1/2 in both WT and ZnT3KO, with similar percent decrease in both genotypes (p-Erk1/2 % increase, WT −35.19 ± 1.41%, *n* = 4; KO, −33.87 ± 3.13%, *n* = 6, *t* = 0.3234 df = 8, *p* = 0.7546; Figure 3A and Supplementary Figure 2A). This indicates that the lack of synaptic Zn^2+^ does not alter NR2B-mediated activation of Erk1/2. Unexpectedly, treatment with KCl+PEAQX increased levels of p-Erk, with a significantly greater increase in the ZnT3KO (p-Erk1/2 % increase, WT, 10.95 ± 6.21%, *n* = 5; KO, 78.90 ± 11.55%, *n* = 5; *t* = 5.181 df = 8, *p* = 0.0008; Figure 3B and Supplementary Figure 2B). This observation suggests that synaptic Zn^2+^ inhibits NR2A-mediated Erk1/2 activation as the removal of Zn^2+^ resulted in increased p-Erk1/2.

### 3.4. BDNF expression is downregulated in ZnT3KO hippocampus

**Figure 4:**
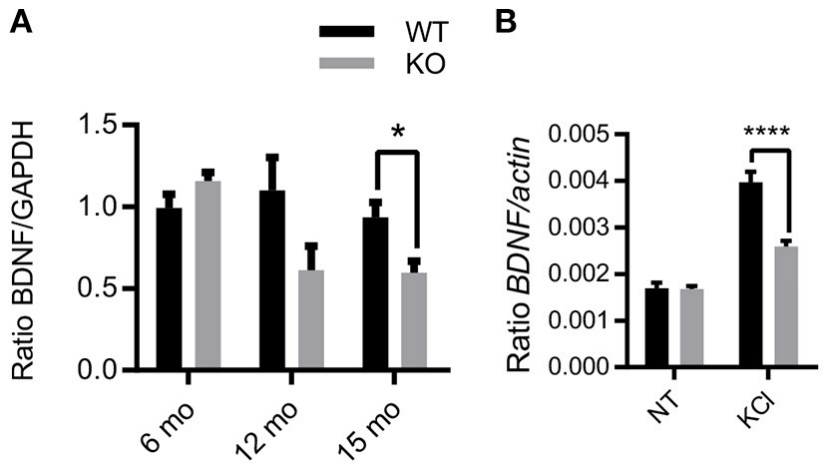
Activity and age-dependent alterations in BDNF expression in ZnT3KO hippocampus. **(A)** Age-dependent alterations in BDNF protein levels were assessed in hippocampus tissue from age-matched WT and ZnT3KO mice by Western blot analysis, finding that ZnT3KO hippocampus has an age-dependent decrease in BDNF protein levels, becoming significant by 15 months of age. **(B)** Activity-dependent increase in BDNF mRNA was assessed through RT-PCR and qPCR analysis in acute hippocampal slices from 3-4-week-old WT and ZnT3KO after 3.5-h incubation in 20 mM KCl. WT, wild type; KO, ZnT3KO; BDNF immunoblots, 6 mo, *n* = 6; 12 mo, *n* = 5; 15 mo, *n* = 6; BDNF mRNA, *n* = 4; NT, no treatment (vehicle); KCl, 3.5 h incubation in 20 mM KCl, *n* = 6; *p < 0.05, ****p < 0.0001; immunoblot values expressed as normalized densitometry relative to GAPDH; BDNF mRNA values expressed as fold change ratio to actin. Data were analyzed by two-tailed unpaired *t*-test. Error bars indicate the mean ± SEM.

Exogenous Zn^2+^ has differential modulation of NMDAR subunits dependent on membrane voltage and Zn^2+^ concentration (24, 25), highlighting the complex interactions of endogenous Zn^2+^ in NMDAR-mediated activation of Erk1/2. To further explore this relationship, we investigated the effects of the removal of synaptic Zn^2+^ on basal and activity-dependent expression of BDNF, which is linked to NMDAR activation of Erk1/2 signaling (34, 61), and is critical for cognitive function and neuronal health. To determine if the removal of synaptic Zn^2+^ alters BDNF expression, we assayed BDNF protein levels in age-matched hippocampus tissue from 6-, 12-, and 15-month-old WT and ZnT3KO mice. Proteins extracted from tissue lysates and analyzed by Western blot normalized to GAPDH showed an age-dependent decrease in BDNF levels becoming significant by 15 months of age (BDNF, WT, 0.93 ± 0.09, *n* = 6; KO, 0.59 ± 0.07, *n* = 6; *t* = 2.932 df = 10, *p* = 0.015; Figure 4A and Supplementary Figure 3). We also investigated whether the increase of BDNF mRNA expression that occurs after depolarization with KCl (62) is altered in ZnT3KO mice using acute hippocampus slices from 3-4-week-old WT and ZnT3KO mice. The slices were incubated for 3.5 h in vehicle or 20 mM KCl, then assayed through RT-PCR and qPCR. The results showed no significant difference in basal levels of BDNF mRNA and a suppressed increase after treatment with KCl in ZnT3KO mice (Ratio *BDNF/actin*, NT, WT 1.91 x 10^−3^ ± 5.28 x 10^−7^, *n* = 6, KO, 2.31 x 10^−3^ ± 1.86 x 10^−6^, *n* = 6, *p* < 0.05; ratio *BDNF/actin* KCl, WT 2.83 ± 0.72, *n* = 6; KO 1.22 ± 0.32, *n* = 6; *t* = 4.93, df = 10, *p* < 0.0001; Figure 4B). These results suggest that ZnT3KO mice have impaired activity-dependent increases in transcription of BDNF mRNA but may also have compensatory mechanisms to maintain BDNF homeostasis that become less effective as they age.

### 3.5. Age-dependent increase of markers of epileptiform activity in ZnT3KO hippocampus

**Figure 5:**
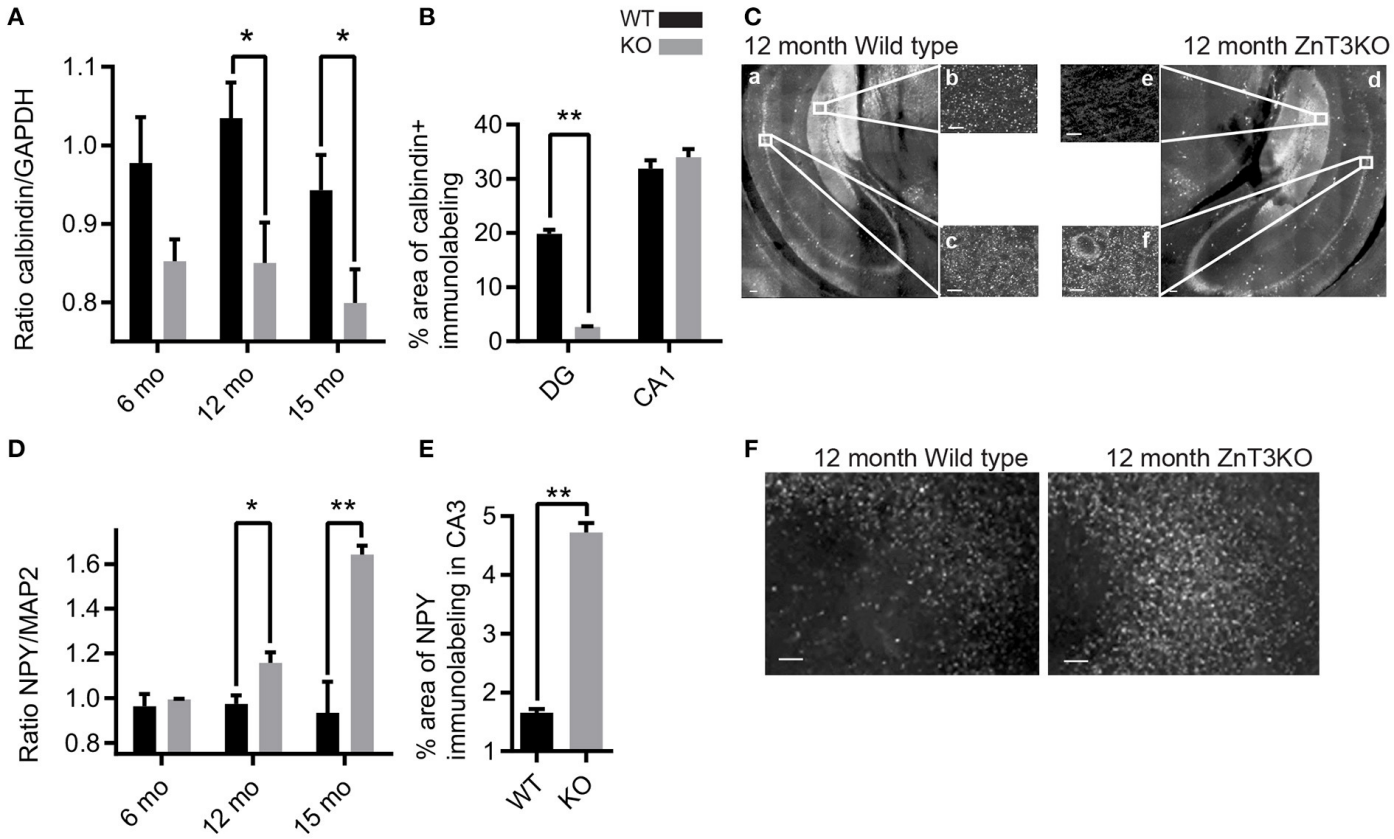
ZnT3KO hippocampus exhibit age-dependent alterations in calbindin and NPY expression that are consistent with alterations resulting from epileptiform activity. **(A)** Western blot analysis of hippocampal tissue shows an age-dependent decrease of calbindin protein levels in ZnT3KO mice. **(B)** Quantification of calbindin immunolabeling shows reduced calbindin in the dentate gyrus, but not in the CA1, region of ZnT3KO hippocampus. **(C)** Representative images of 12-month-old ZnT3KO and WT mice quantified in **(B)**. **(D)** Dot blot analysis of hippocampal tissue shows an age-dependent increase in NPY protein levels in ZnT3KO mice. **(E)** Quantification of NPY immunolabeling shows NPY expression is increased in the CA3 region of ZnT3KO hippocampus. **(F)** Representative images of 12-month old ZnT3KO and WT hippocampus quantified in **(E)**. WT, wild type; KO, ZnT3KO; calbindin immunoblots, 3 and 6 mos. *n* = 5, 12 mo. *n* = 8, 15 mo. *n* = 7; NPY dot blots, 3 and 15 mos. *n* = 4, 6 mo. *n* = 3, 12 mo. *n* = 5; scale bars a and d, 100 μm; b, c, e, and f, 10 μm; *p < 0.05, **p < 0.001; two fields from each subject was imaged at 63x magnification, quantified using the Axiovision (Zeiss) automated measurement program and the percent area calculated. Immunoblot values expressed as normalized densitometry relative to GAPDH or MAP2. Data were analyzed by two-tailed unpaired *t*-test. Error bars indicate the mean ± SEM.

ZnT3KO mice exhibit enhanced susceptibility to induced seizures, suggesting that the lack of synaptic Zn^2+^ causes elevated neuronal activity that reaches seizure threshold with less stimulation, or results in reduced capacity to inhibit excessive activity. Consequently, we investigated the possibility of epileptiform activity in ZnT3KO hippocampus using the biochemical and morphological markers of decreased calbindin expression in the dentate gyrus, increased neuropeptide Y (NPY) expression in the CA3, and aberrant mossy fiber sprouting into the molecular layer of the dentate gyrus. Our results show that ZnT3KO hippocampus has an age-dependent reduction in calbindin protein levels (12 mo., WT, 1.04 ± 0.05, KO, 0.85 ± 0.05, *n* = 8, *t* = 2.697 df = 14, *p* = 0.0173; 15 mo., WT 0.94 ± 0.04, *n* = 7, KO 0.80 ± 0.04, *n* = 7, *t* = 2.314 df = 12, *p* = 0.0392; Figure 5A and Supplementary Figure 4A), with a larger percent decrease of calbindin immunoreactivity in the dentate gyrus (WT, 19.85 ± 0.75, *n* = 2, KO 2.62 ± 0.15, *n* = 2, *t* = 22.36 df = 2, *p* = 0.002) compared with CA1 (WT, 31.9 ± 1.5, *n* = 2, KO, 33.98 ± 1.5, *n* = 2, *t* = 0.9843, df = 2; Figures 5B, C). We also found an age-dependent increase in NPY protein levels in ZnT3KO hippocampus (12 mo., WT, 0.97 ± 0.04, *n* = 5, KO 1.16 ± 0.05, *n* = 5, *t* = 3.017, df = 8, *p* = 0.0166; 15 mo., WT, 0.94 ± 0.14, *n* = 4, KO, 1.64 ± 0.04, *n* = 4, *t* = 4.902, df = 6, *p* = 0.0027; Figure 5D and Supplementary Figure 4B), particularly in CA3 region (12 mo., WT, 1.66 ± 0.06. *n* = 2, KO, 4.72 ± 0.16, *n* = 2, *t* = 17.86, df = 2, *p* = 0.0031; Figures 5E, F).

**Figure 6:**
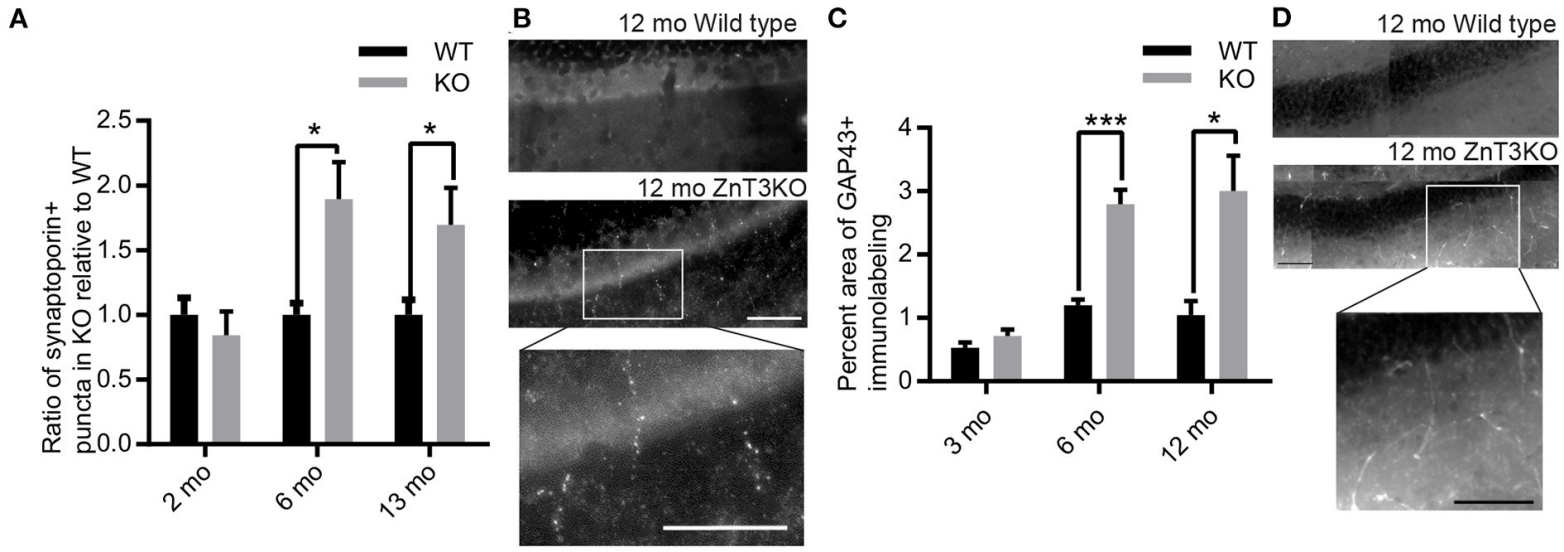
Aberrant, age-dependent mossy fiber sprouting in ZnT3KO hippocampus. **(A)** Quantification of synaptoporin puncta shows an age-dependent increase in mossy sprouting in the molecular layer of the dentate gyrus in ZnT3KO hippocampus. **(B)** Representative image of synaptoporin immunolabeling quantified in A and magnification of KO inset box. **(C)** Quantification of the percent area of GAP43 immunolabeling in the molecular layer of the dentate gyrus shows an age-dependent increase in ZnT3KO hippocampus. **(D)** Representative image of GAP43 immunolabeling quantified in C and magnification of KO inset box. WT, wild type; KO, ZnT3KO; synaptoporin, 6 mo. *n* = 10, 13 mo. WT *n* = 8, KO *n* = 11; scale bars 100 μm; *p < 0.05, ***p < 0.0002; three fields from each subject were imaged at 63X, puncta counted by an experimenter blinded to subject conditions and values expressed as fold change relative to WT puncta for each subject; GAP43, *n* = 5; ten fields from each subject were imaged at 63X, quantified using the Axiovision (Zeiss) automated measurement program and the average percent area calculated. Data were analyzed by two-tailed unpaired *t*-test. Error bars indicate the mean ± SEM.

Mossy fiber sprouting is commonly identified in Zn^2+^-rich mossy fiber tracts using Timm's staining to label Zn^2+^. However, ZnT3KO animals have negligible Zn^2+^ levels in mossy fibers. Thus, we used two different markers to visualize aberrant mossy fiber sprouting: synaptoporin, a synaptic protein preferentially expressed in mossy fibers, and GAP-43, a growth-associated protein found in growth cones of sprouting axons. We found an age-dependent increase in synaptoporin puncta counts expressed as a ratio of ZnT3KO relative to WT (6 mo., WT, 1.00 ± 0.09, *n* = 8, KO, 1.89 ± 0.29, *n* = 8, *t* = 2.975, df = 14, *p* = 0.01; 13 mo., WT, 1.00 ± 0.11, *n* = 8, KO, 1.69 ± 0.29, *n* = 7, *t* = 2.366, df = 13, *p* = 0.0342; Figures 6A, B) and GAP-43 percent area (6 mo., WT, 1.19 ± 0.09, *n* = 5, KO, 2.79 ± 0.23, *n* = 5, *t* = 6.441, df = 8, *p* = 0.0002; 12 mo., WT, 1.17 ± 0.27, *n* = 5, KO, 3.00 ± 0.56, *n* = 5, t = 2.956, df = 8, *p* = 0.0183; Figures 6C, D), in the molecular layer of the dentate gyrus in ZnT3KO relative to WT mice. Together, these results provide biochemical and morphological evidence of age-dependent epileptiform activity which becomes evident beginning at 6 months of age in ZnT3KO mice.

### 3.6. Increased neuronal spiking activity in the hippocampus of young ZnT3KO mice

**Figure 7:**
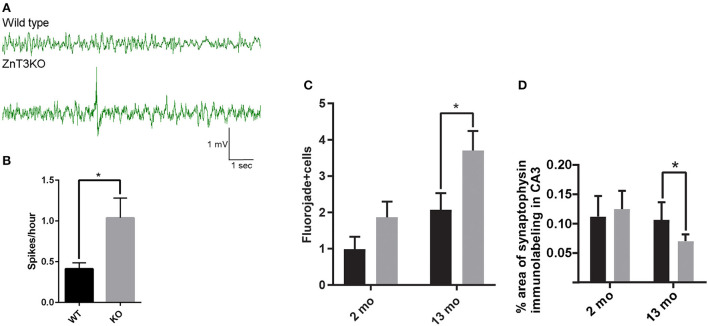
ZnT3KO mice exhibit elevated epileptiform spiking activity, fluorojade+ cells, and reduced synaptophysin expression. **(A)** Representative EEG of 2-month-old WT and ZnT3KO shows spiking epileptiform activity in ZnT3KO. **(B)** 2-month-old ZnT3KO have an elevated rate of spikes per hour. n = 3. EEG data during movement detected by implanted Neurologger recording device was manually removed and raw EEG data was bandpass filtered from 1 to 80 Hz. Spike threshold was a peak amplitude 2.5X greater than the average baseline. **(C)** Image analysis of immunolabeling in hippocampus CA3 region shows an age-dependent decrease in synaptophysin protein levels in ZnT3KO. 2 mo: WT, *n* = 8, KO, *n* = 9; 13 mo: *n* = 6; scale bars 20 μm; one field from each subject was imaged at 63X, quantified using the Axiovision (Zeiss) automated measurement program and the average percent area calculated. **(D)** Quantification of Fluorojade+ cells counted in the CA3 region of the hippocampus show that there is an increased number in 13-month-old KO. 2 mo: WT, *n* = 11, KO, *n* = 9; 13 mo. WT, *n* = = 9, ZnT3KO *n* = 11; the CA3 region was imaged at 20X magnification using the Mosaic tool in Axiovision to image and stitch together adjacent fields and the number of Fluorojade positive cells were counted by an experimenter blinded to subject conditions. *p < 0.05. Data were analyzed by two-tailed unpaired *t*-test. Error bars indicate the mean ± SEM.

Evidence of age-dependent increases in markers of epileptiform activity invited investigation to determine the age of onset of aberrant EEG in ZnT3KO mice. Using a wireless EEG recording device, we analyzed epileptiform spiking activity in freely moving 2-month-old mice in their home cages during ≈24-h recording sessions, finding a 147% increase in the rate of spikes per hour (WT, 0.40 ± 0.06, *n* = 3; KO, 0.99 ± 0.19, *n* = 3; *t* = 2.864, df = 4, *p* = 0.0457) in ZnT3KO mice (Figures 7A, B). The presence of increased epileptiform spiking in 2-month-old ZnT3KO is possibly the genesis or precursor of more extensive neuronal hyperactivity resulting in markers of epileptiform activity by 6 months of age.

### 3.7. Age-dependent increase in markers of neurodegeneration in the hippocampus of ZnT3KO mice

Altered expression and activation of neurotrophic proteins and epileptiform activity may result in synaptic loss and neurodegeneration. We quantified synaptic density using synaptophysin immunolabeling and degenerating neurons through Fluorojade labeling. Image analysis showed a significant reduction in synaptic density in the CA3 region of 13-month-old ZnT3KO mice determined by percent area of synaptophysin immunolabeling (WT, 0.167 ± 0.034, *n* = 6; KO, 0.070 ± 0.011; *n* = 6; *t* = 2.687, df = 10, *p* = 0.0228; Figure 7C) and frequent degenerating neurons *in the CA3 region of the hippocampus* in 13-month old ZnT3KO mice (WT, 2.08 ± 0.46, *n* = 11; KO, 3.70 ± 0.54; *n* = 11; *t* = 2.87, df = 20, *p* = 0.0095; Figure 7D), with no significant difference found in 2- or 3-month-old ZnT3KO mice, indicating that the lack of synaptic Zn^2+^ leads to age-dependent synaptic and neuronal loss.

## 4. Discussion

ZnT3KO mice were initially reported to have normal cognition and sensorimotor responses, suggesting that synaptic zinc has minimal neuromodulatory effects or its effects are easily compensated for. Other studies have found that ZnT3KO mice develop cognitive deficits and neurodegenerative biochemical alterations as they age. These studies indicate that the genetic removal of synaptic Zn^2+^, while not directly causing cognitive impairment, disrupts the normal role of Zn^2+^ in neurotransmission, leading to neurodegenerative processes as the mice age. Zn^2+^ is a modulator of NMDAR responses in both neurotrophic and neurodegenerative pathways (29) and also modulates the development of neuronal hyperactivity and seizures (38, 39). We used acute hippocampal slice cultures from juvenile ZnT3KO mice to assess basal protein expression and protein activation modulated by NMDARs, identifying altered expression and activation of proteins that have been demonstrated to be modulated by the addition or chelation of Zn^2+^ in cultures of dissociated neurons (3, 19, 25, 40, 54). We used EEG recordings from young ZnT3KO to assess neuronal hyperactivity before the onset of cognitive deficits, finding that 2-month-old ZnT3KO mice have elevated EEG spiking activity, which is consistent with findings of a lower threshold for induced seizures in ZnT3KO (36, 37). We used aged ZnT3KO to assess age-dependent alterations in cognition, protein expression modulated by NMDARs, and biochemical and brain cytoarchitecture consistent with epileptiform activity. We found a profile of progressive cognitive impairment beginning at 3 months of age and becoming profound by 6 months, age dependent decline in BDNF, a neurotrophic protein differentially regulated by NMDARs, and an age dependent increase in markers of epileptiform activity.

Besides structural and synaptic zinc, there is another pool of zinc in the brain: a transient pool referred to as tonic, or “free” zinc, which is unbound Zn^2+^ present in the cytosol or interstitial fluid yet to be sequestered by Zn^2+^-binding proteins (1). Zinc homeostasis appears to be disrupted with age (12, 63). Our findings are consistent with recent reports which indicate that both synaptic and tonic Zn^2+^ can modulate neurotransmission (6, 35). Basal expression of Erk1/2 and AKT after treatment with vehicle, a condition mimicking the effects of tonic Zn^2+^, are reduced in the ZnT3KO hippocampus, indicating that elimination of synaptic Zn^2+^ may reduce the pool of tonic Zn^2+^ available to regulate protein expression. The activity-dependent increase in phosphorylated Erk1/2 and BDNF mRNA levels are also reduced in ZnT3KO mice, demonstrating direct effects of the release of synaptic Zn^2+^ on protein regulation. The reduced activation of Erk1/2 and no change in activation of AKT in ZnT3KO mice indicates that synaptic Zn^2+^ released following depolarization modulates the Raf/MEK/Erk signaling cascade, but not the PI3K/AKT pathway, and supports the recent observation that synaptic Zn^2+^ does not promote TrkB activation *in vivo* (64, 65).

NMDAR-mediated activation of Erk1/2 has differential regulation of BDNF expression that is dependent on NMDAR location, subunit composition, and intensity of neuronal stimulation (29, 66). To investigate how synaptic Zn^2+^ influences NMDAR activity, we used antagonists of the NR2A and NR2B subunits, finding that the NR2B antagonist ifenprodil reduces p-Erk1/2 similarly in both genotypes, indicating that synaptic Zn^2+^ does not modulate NR2B-mediated activation of Erk1/2. Conversely, the NR2A antagonist PEAQX restores p-Erk in ZnT3KO, indicating that Zn^2+^ modulation of NR2A inhibits activation of Erk1/2. Conflicting roles for the NR2A subunit in Erk1/2 activation have been reported (32, 67), suggesting that NR2A-mediated activation of Erk1/2 is dependent on variable factors. Zinc has biphasic regulation of NMDARs, facilitatory at low levels and repressive at high levels (6, 35, 68), with high affinity for NR2A subunits (27), and tonic inhibition of NR2A (69). Zinc inhibition of NR2A at high levels of synaptic activity indicate a mechanism for zinc to attenuate excessive excitatory neurotransmission.

Zn^2+^ has been demonstrated to reduce susceptibility to hyperexcitability and seizure (36–39) and potentiate inhibitory glycine receptors at mossy fiber axon terminals (70, 71). Removal of Zn^2+^ inhibition would tip the balance of neurotransmission toward excessive excitation. Markers of epileptiform activity were assessed in hippocampal tissue from age-matched cohorts of WT and ZnT3KO mice. We found an increase in mossy fiber sprouting at 6 months of age in ZnT3KO, when cognitive impairment becomes profound in this genotype, and biochemical markers of epileptiform activity apparent at 12 months of age in ZnT3KO mice. Synaptic Zn^2+^ is released in the CA3 and the dentate gyrus (DG) of the hippocampus under both normal and hyperactive conditions, so the removal of Zn^2+^ would impact those regions significantly. Zn^2+^-enriched mossy fibers arise from granule cells of the DG and normally project to the CA3 region, where they terminate to form synapses with CA3 pyramidal cells and GABAergic interneurons, and also form small terminals that synapse with GABAergic cells in the hilus of the DG (72). Neuronal hyperactivity in the hippocampus triggers mossy fibers to sprout, forming aberrant excitatory collaterals terminating in the inner molecular layer of the DG, causing excessive excitation of adjacent granule cells that project into the hilus of the DG (73), and to form recurrent excitatory collaterals that generate a feed-forward circuit, increasing neuronal excitability and resulting in increased hyperactivity (74–76). Hyperactivity also stimulates newborn granule cells from adult neurogenesis to develop in abnormal locations in the hilus and receive mossy fiber input (77). Thus, the lack of Zn^2+^ inhibition in the hippocampus can trigger a cascade of neuronal hyperexcitability leading to epileptiform activity and seizures. NPY suppresses epileptiform activity and its expression is upregulated in response to seizures (78, 79). Consequently, our observation of age-dependent increased NPY expression in the CA3 is accordant with the removal of Zn^2+^ enhancement of inhibitory response, as is our observation of age-dependent decrease of calbindin, a cytoplasmic protein that buffers intraneuronal calcium and is downregulated by seizures in the DG region but not in CA1 (80). Neurodegenerative markers resulting from the cumulative effects of epileptiform activity and altered neuronal signaling were assessed in hippocampal tissue from age-matched cohorts of WT and ZnT3KO mice. We found a reduction in the synaptic protein synaptophysin and an increase in Fluorojade labeling, two common markers of neurodegeneration, to be most pronounced in the CA3 region of aged ZnT3KO mice, indicating that the lack of synaptic Zn2+ has a profound impact in that hippocampal region.

Aberrant neuronal hyperactivity and hippocampal circuit reorganization has also been found in transgenic mice overexpressing hAPP [(81), reviewed in (82)], and treatment with an anti-seizure drug reverses hippocampal remodeling, synaptic dysfunction and cognitive deficits in hAPP mice (83). In humans, patients with amnestic mild cognitive impairment (aMCI, often a precursor to AD) who also have epilepsy develop symptoms of cognitive decline earlier than aMCI patients without epilepsy, and AD patients with subclinical epileptiform activity also have an earlier onset of cognitive decline (84). Recent research has detected clinically silent seizures and epileptiform spiking in the hippocampi of AD patients (85) and patients with some forms of familial AD are at greater risk of suffering seizures (86). Also, treatment with an anti-seizure drug improves cognition in aMCI patients (87–89). These observations, along with our findings, indicate that clinically silent seizures and epileptiform activity are present early in and contribute to the progression of cognitive decline, and also suggest the possibility that AβO interference with Zn^2+^ signaling promotes cognitive decline through increasing neuronal hyperactivity.

Our findings provide insight into why ZnT3KO mice begin life phenotypically normal but develop cognitive impairment and neurodegeneration as they age. We found alterations in protein expression, neuronal signaling, and EEG spiking in ZnT3KO mice before the onset of cognitive impairment, which indicates that while these alterations are not sufficient to impair cognition, they can contribute to neurodegeneration. Zn^2+^ has been shown to have biphasic regulation of NMDARs that is dependent on variable factors (29, 66), as has the expression of BDNF mediated by Erk1/2 activation through NMDARs (34, 61). Using KCl application to hippocampal slice cultures to stimulate neurotransmission, we observed that activity-dependent activation of Erk1/2 and expression of BDNF mRNA is greater in WT than in ZnT3KO, suggesting that Zn^2+^ is a modulator of Erk1/2-mediated BDNF expression. We also observed that Zn^2+^ inhibits NR2A-containing NMDARs, indicating that Zn^2+^ inhibition of NR2A in WT may increase BDNF mRNA expression. These observations invite future investigation of activity-dependent expression of BDNF mRNA in the presence of NR2A antagonists in ZnT3KO to clarify the role of Zn^2+^ inhibition of NR2A-containing NMDARs in BDNF expression. We also found markers of epileptiform activity beginning with the onset of profound cognitive impairment and subsequent decreased BDNF expression and synaptic density and increased neurodegeneration in aged ZnT3KO mice, advocating for compensatory mechanisms that are eventually overcome by increasing neurodegeneration.

These observations lead us to propose that the cumulative effects of interference with synaptic Zn^2+^ modulation of neurotransmission triggers neurodegenerative pathways and accelerates neuronal excitotoxicity that ultimately disrupt synaptic signaling and induce significant neurodegeneration in an age dependent manner. ZnT3 levels decline with age in mice, rats and humans, and decline even further in AD (12, 43, 90), suggesting that disruption of zinc homeostasis contributes to AD pathology. The source of cognitive impairment in AD is not well-established. Cognitive decline correlates with soluble Aβ (91) as well as with tangle density, but that does not demonstrate the causative role of tau or Aβ in AD. Multiple studies show the neurotoxic effects of Aβ (92, 93), providing a pathway for cognitive decline. More recently, positive clinical trial results show a delay in cognitive decline in patients with immunotherapy targeting Aβ (Lecanemab), not tau. In addition, multiple evidences show that tau pathology is likely initiated by Aβ meaning that targeting Aβ may still affect tau pathology (94–97). Together these findings support further research into targeted interventions to reduce AβO formation and activity and to preserve synaptic zinc homeostasis in the brain of affected patients.

## Data availability statement

The raw data supporting the conclusions of this article will be made available by the authors, without undue reservation.

## Ethics statement

The animal study was reviewed and approved by IACUC, University of California-Irvine.

## Author contributions

EV, MM, KS, RB, and SC-S performed experiments, analyzed results, and prepared figures. EV and JB designed experiments and interpreted results. EV, MM, and JB wrote and edited manuscript. All authors contributed to the article and approved the submitted version.
