# Supplementary material for: Genetic removal of synaptic Zn2+ impairs cognition, alters neurotrophic signaling and induces neuronal hyperactivity
Source: Front Neurol. 2023 Jan 20;13:882635. doi: 10.3389/fneur.2022.882635 (PMC9895830; doi:10.3389/fneur.2022.882635)
Supplement: Supplementary file 1 [file Data_Sheet_1.pdf]

Supplementary Figures

Figure S1

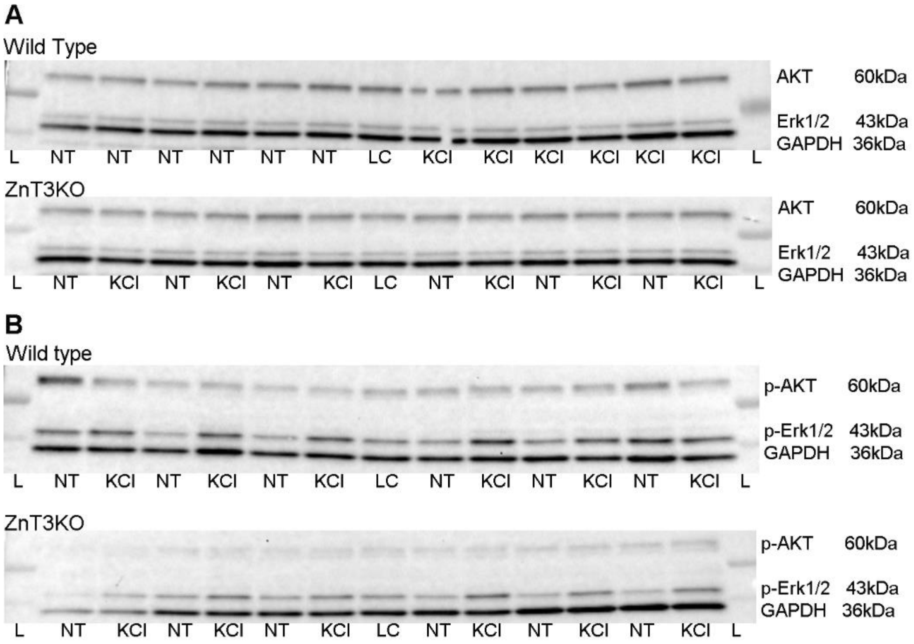

Figure S1. Western blot of wild type and ZnT3KO hippocampal homogenates quantified in Figures 2 and 3. (A) Representative images of AKT and Erk1/2 immunoreactivity quantified in Figure 2A. **(B)** Representative images of p-AKT and p-Erk1/2 immunoreactivity quantified in Figure 2C and Figure 3. L, molecular weight ladder markers (L) at 36kDa and 50kDa; NT, no treatment; KCl, 10-minute incubation in 20mM KCl; LC, loading control.

Figure S2

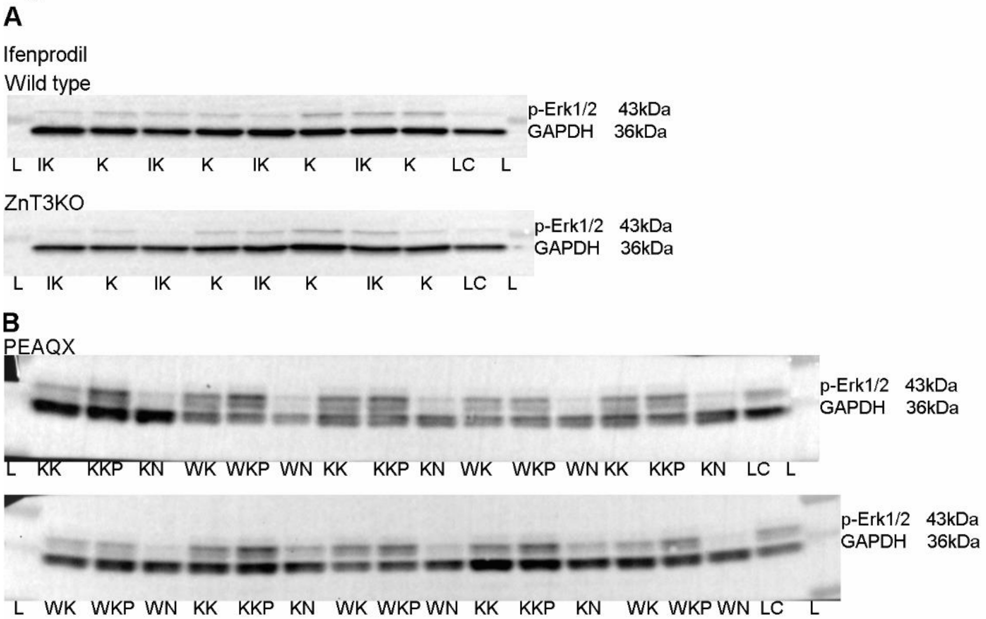

Figure S2. Western blot of wild type and ZnT3KO hippocampus homogenates quantified in Figure 3. **(A)** Representative images of p-Erk1/2 immunolabeling quantified in Figure 3A. IK, 10-minute incubation in ifenprodil/20mM KCl; K, 10-minute incubation in 20mM KCl; L, ladders at 36kDa and 50kDa; LC, loading control. **(B)** Representative images of p-Erk1/2 immunolabeling quantified in Figure 3B. KK, ZnT3KO/10-minute incubation in 20mM KCl; KKP, ZnT3KO/10-minute incubation in PEAQX/20mM KCl; KN, ZnT3KO/no treatment; WK, wild type/10-minute incubation in 20mM KCl; WKP, wild type/10-minute incubation in PEAQX/20mM KCl; WN, wild type/no treatment; L, molecular weight ladder markers at 36kDa and 50kDa; LC, loading control.

Figure S3

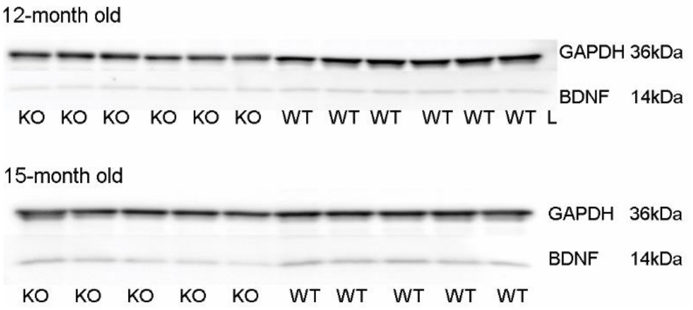

Figure S3. Western blot of wild type and ZnT3KO tissue homogenates quantified in Figure 4A. Representative images of BDNF immunolabeling quantified in Figure 4A.

Figure S4

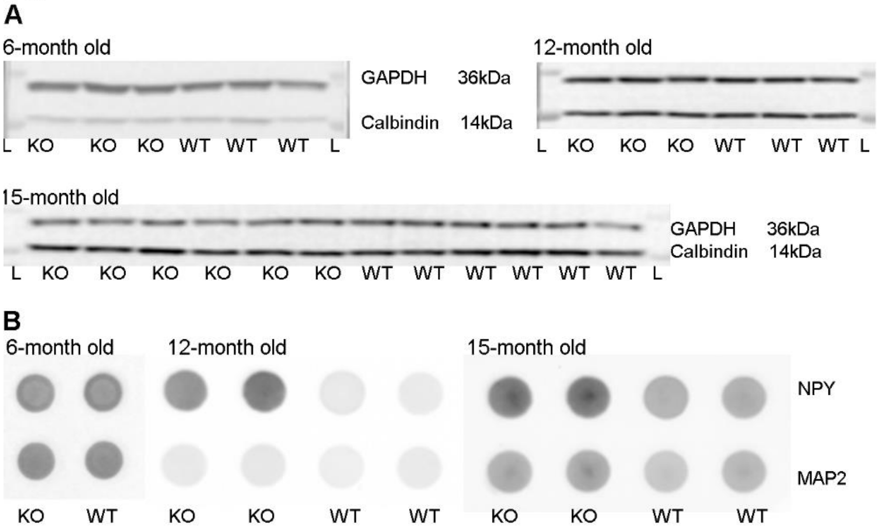

Figure S4. Immunoblot of wild type and ZnT3KO tissue homogenates quantified in Figure 5. (A) Representative images of calbindin immunolabeling quantified in Figure 5A. **(B)** Representative images of NPY immunodots quantified in Figure 5D. Molecular weight ladder markers (L).

Figure S5

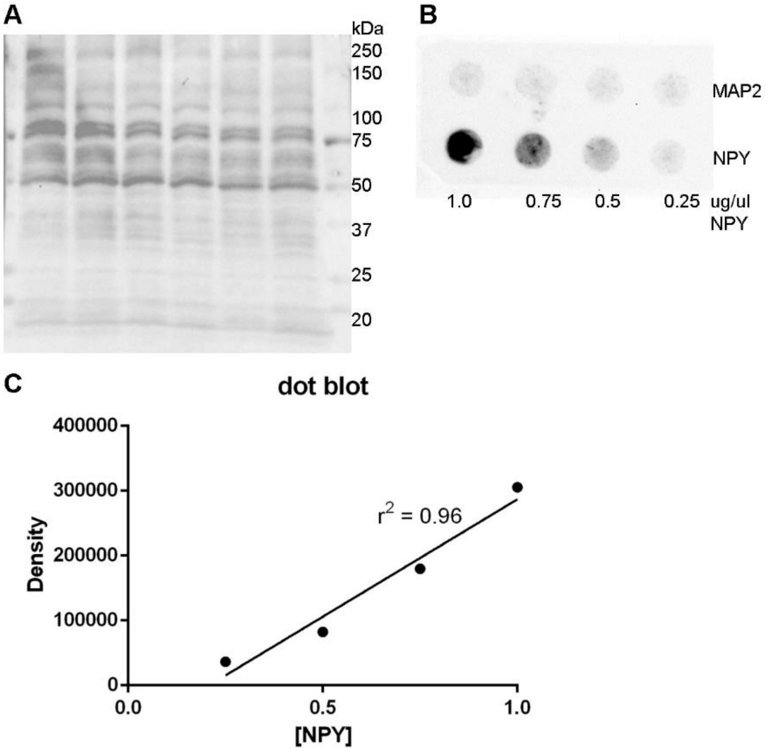

Figure S5. NPY protein forms aggregates during gel electrophoresis. (A) The 11kDa NPY forms aggregates resulting in multiple bands. **(B)** Dot blot of known concentrations of purified NPY standard. **(C)** Standard generated curve generated using NPY dot blot data.
